# Community Structure, Assembly and Interactions of *Nitrospira* Nitrite-Oxidizing Bacteria in Sediments of the Eastern China Marginal Seas

**DOI:** 10.3390/microorganisms13051112

**Published:** 2025-05-12

**Authors:** Hao Dong, Hui He, Min Wang

**Affiliations:** 1 College of Marine Life Sciences, Key Laboratory of Evolution & Marine Biodiversity (Ministry of Education) and Institute of Evolution & Marine Biodiversity, Frontiers Science Center for Deep Ocean Multispheres and Earth System, Ocean University of China, Qingdao 266003, China; donghao4684@stu.ouc.edu.cn (H.D.); mingwang@ouc.edu.cn (M.W.); 2 Haide College, Ocean University of China, Qingdao 266100, China; 3 UMT-OUC Joint Academic Centre for Marine Studies, Ocean University of China, Qingdao 266003, China

**Keywords:** *Nitrospira*, community characteristics, interaction, eastern China marginal seas, marine sediments

## Abstract

Nitrite oxidation, a pivotal process in the nitrogen cycle driven by microorganisms, is primarily carried out by nitrite-oxidizing bacteria (NOB). While extensive studies on *Nitrospira* have been conducted in terrestrial habitats, knowledge of *Nitrospira* in marine sediments remains limited. Therefore, we employed high-throughput sequencing analysis to systematically explore the community structure, assembly processes and potential interactions of *Nitrospira* in the eastern China marginal seas. Our results exhibit pronounced spatial heterogeneity in *Nitrospira* communities across seas. Widespread distribution of *Nitrospira* taxa was observed, with *Nitrospira* lineage II emerging as the most important group in this study. Based on the neutral community model (NCM), normalized stochasticity ratio (NST) and beta nearest-taxon-index (βNTI) analysis, deterministic processes predominantly shaped the community assembly of *Nitrospira*. Complex interspecies interactions among *Nitrospira* were observed with molecular ecological network analysis, and the community in the East China Sea showed the highest complexity, while the community displayed the greatest stability in the South Yellow Sea. In addition, the *Nitrospira* communities were notably influenced by geographic distances and environmental factors, including salinity, temperature, dissolved oxygen concentration and dissolved inorganic nutrient concentration. These results may expand our understanding of *Nitrospira* in marine environments and enhance our insights into the marine nitrogen cycle.

## 1. Introduction

The nitrogen cycle is important to global biogeochemical processes and primarily mediated by microorganisms. Nitrification is a critical process that connects the most oxidized and reduced forms of inorganic nitrogen, which maintain the nitrogen balance on a global scale [1,2]. Classical nitrification involves two sequential steps, that is, ammonia oxidation mediated by ammonia-oxidizing bacteria (AOB) and archaea (AOA), as well as nitrite oxidation driven by nitrite-oxidizing bacteria (NOB) [3,4,5]. Currently, NOB are the only microorganisms that can convert nitrite to nitrate under aerobic conditions [6]. Among the seven recognized genera of NOB, *Nitrospira* exhibits the widest distribution, strongest substrate affinity, highest diversity and greatest phylogenetic richness, making it crucial in diverse ecosystems, including marine sediments [7,8,9].

*Nitrospira* is classified into six sublineages based on the current phylogenetic analysis and is prevalent in diverse habitats, including wastewater treatment systems, freshwater and forest soil [10,11,12]. Its ubiquity and essential role in nitrogen transformation make it a research hot spot in the nitrogen cycle [7,13]. Traditionally, *Nitrospira* was regarded as a strict obligate chemolithoautotroph [14]. However, recent discoveries have challenged this view. Some *Nitrospira* strains can utilize simple organic carbon sources such as glycerol, acetate and formate [15,16,17]. Certain strains can express urease to convert urea to ammonium [14]. Moreover, the discovery of a complete ammonia oxidizer (comammox) *Nitrospira* within lineage II, which is crucial for nitrogen removal to mitigate eutrophication and other related ecological issues in marine ecosystems, was a significant breakthrough in research on the nitrogen cycle [18,19]. Comammox can also produce nitrous oxide as a byproduct, which leads to the greenhouse effect and the depletion of the ozone layer [20,21]. These findings emphasize the significance of *Nitrospira*; however, our understanding of *Nitrospira* in marine sediments remains limited.

The eastern China marginal seas (ECMSs), which include the Bohai Sea (BS), the Yellow Sea (YS) and the East China Sea (ECS), receive approximately 1.5 × 10^9^ tons of terrestrial sediments annually from major rivers such as the Yangtze River and the Yellow River, accounting for nearly 10–12% of global marginal sea inputs [22,23,24]. These regions also face diverse pollution pressures from urbanization and industrial and agricultural development [25], resulting in microbial pollution loads being 15–30% higher than in other marine sedimentary habitats. In addition, these pollution pressures have led to a 20–35% increase in heterotrophic bacterial abundance and altered N:P ratios [26,27]. These factors collectively influence microbial community structure and functions in the ECMSs, thereby impacting marine nutrient cycles. Hence, the ECMSs have long been a focus of research on marine nutrient cycles. A considerable amount of research has been carried out to explore the community composition and phylogenetic characteristics of certain microorganisms related to the nitrogen cycle, such as AOA, AOB, anammox bacteria and denitrifiers, in these regions [28,29,30,31]. Previous studies have characterized the community composition of comammox *Nitrospira* lineage II in sediments of the ECMSs [32]. However, our understanding of *Nitrospira*, including other lineages, particularly their community assembly and interactions, remains limited in this region.

To address this gap, sediment samples from the ECMSs were collected to explore the community structure, assembly and interactions of *Nitrospira* through high-throughput sequencing analysis in the present study. The primary objectives were to (1) determine the diversity, composition and phylogeny of *Nitrospira* communities, along with their potential factors; (2) examine the relative roles of deterministic and stochastic processes in the community assembly of *Nitrospira*; and (3) explore the potential interactions within *Nitrospira* communities and evaluate their community stability in sediments of the ECMSs. The results contribute to a more comprehensive understanding of the nitrogen cycle in oceanic sedimentary habitats and provide valuable insights into the adaptive strategies of marine microorganisms to environmental changes.

## 2. Materials and Methods

### 2.1. Sediment Sample Collection and Environmental Characterization

**Figure 1:**
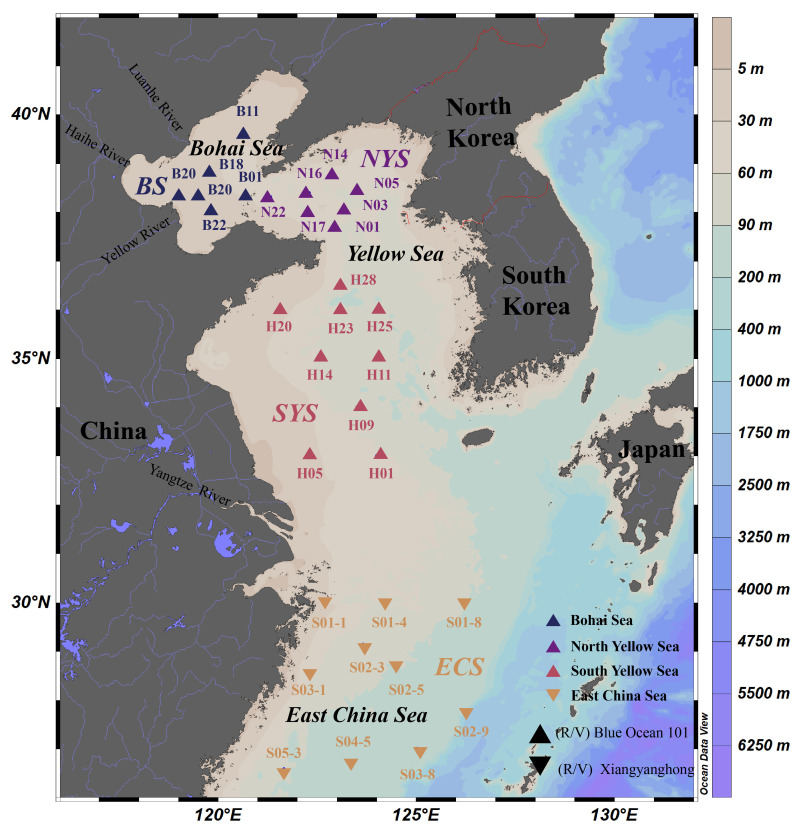
Locations of sampling stations in the eastern China marginal seas in this study.

**Figure 2:**
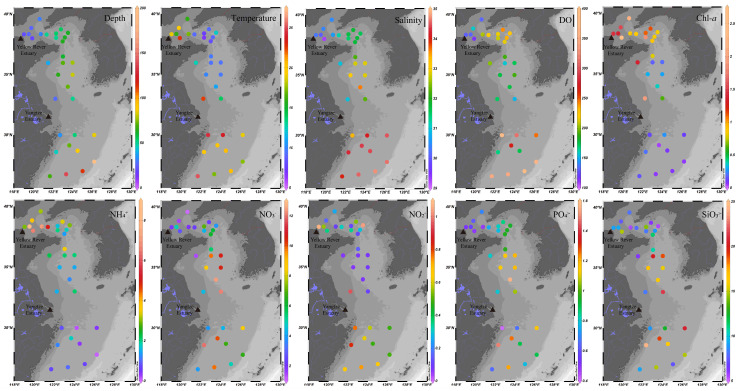
Environmental factors of sampling stations.

A total of 32 surface sediment samples were collected from the ECMSs during two oceanographic surveys. The sampling stations are depicted in Figure 1. Samples from the BS and the YS were taken from July to August 2023 on the research vessel (R/V) Blue Ocean 101, and samples from the ECS were gathered in October 2023 on the R/V Xiangyanghong 18. To ensure consistency in the sampling methodology, all sediment samples were collected with the same model of box corer during both expeditions, followed by identical protocols for the collection and preservation of the sediments. For environmental characterization, bottom seawater at each station was obtained in this study. Temperature, salinity, dissolved oxygen (DO) concentration and depth were recorded in situ with a conductivity–temperature–depth (CTD) multi-parameter profiler (Seabird, Bellevue, WA, USA). The chlorophyll *a* (chl *a*) content was measured with a TD10-AU fluorometer (Turner Designs, San Jose, CA, USA) [33]. After filtering the bottom water through a 0.45 μm membrane, the concentrations of dissolved inorganic nutrients were determined using a Quattro39 nutrient autoanalyzer (Seal Analytical, Norderstedt, Germany). The results of the environmental factors are presented in Figure 2.

### 2.2. DNA Extraction and High-Throughput Sequencing Analysis

Sedimentary DNA was extracted via a DNeasy PowerSoil Kit (Qiagen, Hilden, Germany) following the standard protocols. After extraction, DNA purity and concentration were assessed via a microspectrophotometer (Aosun Nano-300, Suzhou, China). Only DNA that met the quality criteria was used as a template for PCR amplification. The *nxrB* gene of *Nitrospira* was amplified using the specific primer pair nxrB169F and nxrB638R, with barcode sequences ligated prior to amplification [34]. The qualified DNA libraries were sequenced with a NovaSeq 6000 platform at Megageno Technology Co., Ltd. (Guangzhou, China).

### 2.3. Sequence Data Processing and Statistical Analysis

Raw data were processed and analyzed using Quantitative Insights Into Microbial Ecology 2 (QIIME2) [35]. The raw sequences were imported into QIIME2 via the import plugin. Primer-trimmed paired-end sequences were merged with the deblur plugin. Next, the merged sequences were further processed to eliminate redundant sequences, and operational taxonomic units (OTUs) were clustered based on a 97% sequence similarity threshold with the vsearch plugin [6,36]. To minimize errors from amplification or sequencing, singleton and doubleton OTUs were discarded [37]. The raw sequencing data were normalized using the feature-table plugin to mitigate biases from uneven sequencing depths across samples. Representative sequences of each OTU were taxonomically classified against the NCBI database. Alpha diversity, including community richness, diversity and evenness, was calculated after sequence homogenization [38]. A phylogenetic tree was constructed with the Mafft alignment and the FastTree method. Non-metric multidimensional scaling (NMDS) analysis based on the Bray–Curtis distance was applied to evaluate the difference of *Nitrospira* communities across the ECMSs. A Mantel test and canonical correspondence analysis (CCA) was conducted to evaluate the effects of geographic distance and environmental factors on the *Nitrospira* communities in this study.

### 2.4. Community Assembly, Co-Occurrence Network and Stability Analysis

Neutral community model (NCM) analysis and the normalized stochasticity ratio (NST), along with the beta nearest taxon index (βNTI) based on the phylogenetic dissimilarity, were utilized to assess the relative importance of stochastic and deterministic processes in the assembly of *Nitrospira* communities using the “minpack.lm”, “NTI”, “HMisc” and “MicEco” packages in R (version 4.1.3) [39,40]. In this study, the co-occurrence network was constructed based on the random matrix theory (RMT) with molecular ecological network analysis (MENA). OTUs with a relative abundance exceeding 0.1% and a significant correlation (*p* < 0.05) with an RMT threshold greater than 0.83 were retained for network analysis [41]. The generated network was visualized, and the topological properties were calculated using Gephi (version 0.9.2) [42]. To assess the stability of *Nitrospira* communities in the ECMSs, robustness and vulnerability analyses were explored with the “robustbase” and “igraph” packages in R [43].

## 3. Results

### 3.1. Nitrospira Community Characteristics

**Figure 3:**
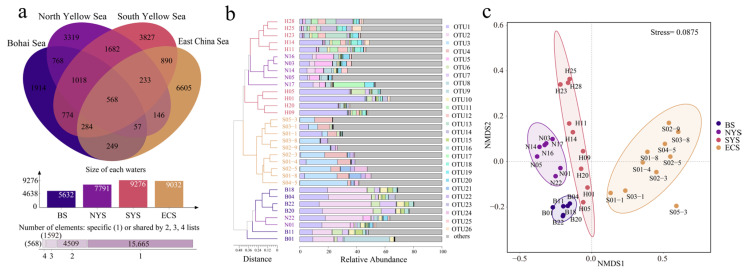
(**a**) Venn diagram of *Nitrospira* communities across the ECMSs. (**b**) Clustering analysis of dominant OTUs with a relative abundance greater than 0.5% in sediments of the ECMSs. (**c**) NMDS analysis of *Nitrospira* communities in sediments of the ECMSs.

A total of 3,548,774 high-quality *nxrB* gene sequences were obtained in this study, with the number of sequences ranging from 56,939 to 195,783 at each station. Normalization was carried out based on the minimum sequence count across samples, and the rarefaction curves suggest that microbial diversity approached a plateau at this threshold. Based on a 97% sequence similarity, 22,334 OTUs were identified, varying from 1101 to 3019 per station. The Venn diagram analysis reveals that 568 OTUs were common across seas, which accounted for 2.5% of the total OTUs in this study (Figure 3a). The ECS exhibited the highest numbers of unique OTUs (6605), followed by the SYS (3827), the NYS (3319) and the BS (1914), implying obvious differences in the composition of *Nitrospira* communities across seas. To further analyze the variations in *Nitrospira* communities, dominant OTUs with a relative abundance greater than 0.5% were chosen for clustering analysis, as shown in Figure 3b. The dominant OTUs from different seas clustered on different branches, suggesting that their community characteristics varied across seas. NMDS analysis further verifies the notable spatial heterogeneity of *Nitrospira* communities in sediments along the ECMSs (*p* < 0.01, Figure 3c).

**Figure 4:**
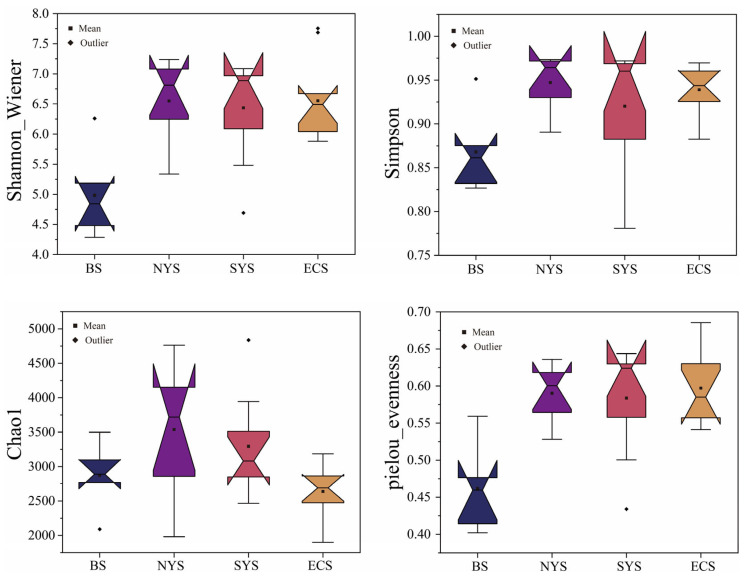
Alpha diversity of *Nitrospira* communities in sediments of the ECMSs.

The coverage of the *Nitrospira* community at each station exceeded 99%, indicating the sequences could appropriately represent the majority of *Nitrospira* taxa in the studied seas. The Chao1, Shannon–Wiener, Simpson and Pielou’s evenness indices, respectively, varied from 1898.08 to 4836.90, 4.29 to 7.76, 0.78 to 0.97 and 0.40 to 0.69 in the present study. As illustrated in Figure 4, the above-mentioned alpha diversity indices peaked in the YS, followed by the ECS and the BS. Notably, the Shannon–Wiener, Simpson and Pielou’s evenness indices exhibited their highest values in the SYS, while the Chao1 index peaked in the NYS.

### 3.2. Phylogenetic Characteristics of Nitrospira

**Figure 5:**
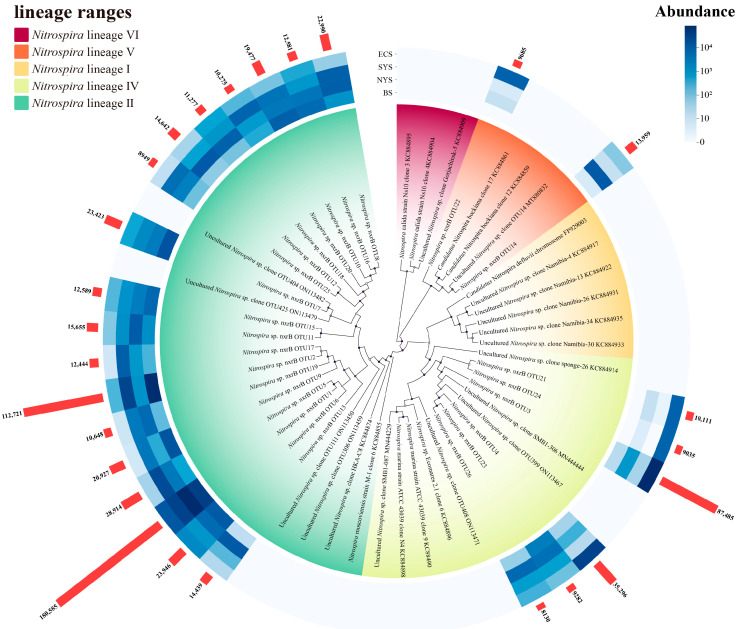
Phylogenetic analysis of dominant *Nitrospira* OTUs in sediments of the ECMSs.

The phylogenetic characteristics of *Nitrospira* were explored by comparing the dominant OTUs (with a relative abundance greater than 0.5%) against the NCBI database, as shown in Figure 5. Consistent with previous studies, the most dominant OTUs primarily belonged to uncultured *Nitrospira* lineages. Three *Nitrospira* lineages were identified among the dominant OTUs, with lineage II being the predominant group (69.23%), followed by lineage IV (23.08%) and lineage V (7.69%), which indicates the significant role of lineage II in *Nitrospira* communities in the studied sediments. Furthermore, distinct distribution patterns of the top three dominant OTUs were observed across different seas. In the BS, the most abundant OTUs (OTU2, OTU1 and OTU7) all belonged to lineage II. Similarly, the top three OTUs (OTU1, OTU12 and OTU13) in the NYS, as well as the top three OTUs (OTU1, OTU2 and OTU5) in the SYS, were affiliated with lineage II. In contrast, the three most abundant OTUs (OTU3, OTU4 and OTU1) in the ECS exhibited a different pattern, with OTU1 belonging to lineage II, while OTU3 and OTU4 were classified into lineage IV. Regional differences were identified in the phylogenetic composition of *Nitrospira* communities across seas, particularly in the ECS, which suggests the spatial heterogeneity of *Nitrospira* communities across the ECMSs.

### 3.3. Effects of Environmental Factors on Nitrospira Communities

**Figure 6:**
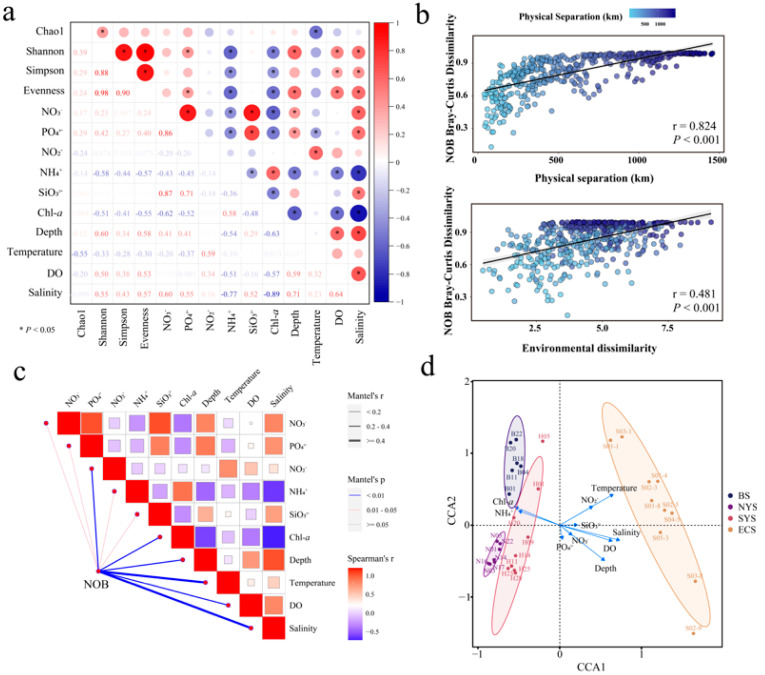
(**a**) Effects of environmental factors on the community alpha diversity of *Nitrospira*. (**b**) Relationship between geographical distances (or environmental factors) and *Nitrospira* communities. (**c**) Effects of environmental factors on the *Nitrospira* community compositions. (**d**) CCA of *Nitrospira* communities in sediments of the ECMSs.

The relationship between environmental factors and community alpha diversity is shown in Figure 6. The Shannon–Wiener index was significantly associated with ammonium concentration (*p* < 0.01), chl *a* content (*p* < 0.01), salinity (*p* < 0.01) and DO concentration (*p* < 0.01) and phosphate concentration (*p* < 0.05). Ammonium concentration (*p* < 0.05), depth (*p* < 0.05), chl *a* content (*p* < 0.05), salinity (*p* < 0.05) and DO concentration (*p* < 0.05) were the main drivers affecting the Simpson index. The Chao1 index was remarkably correlated with temperature (*p* < 0.01). Pielou’s evenness index exhibited notable correlations with depth (*p* < 0.01), ammonium concentration (*p* < 0.01), chl *a* content (*p* < 0.01), salinity (*p* < 0.01), DO concentration (*p* < 0.01) and phosphate concentration (*p* < 0.05).

Both geographical distances (*p* < 0.01) and environmental factors (*p* < 0.01) notably correlated with the *Nitrospira* communities in this study, according to the Mantel test and linear regression analysis (Figure 6b). The most influential environmental factors included temperature (*p* < 0.01), salinity (*p* < 0.01), nitrite concentration (*p* < 0.01), depth (*p* < 0.01), DO concentration (*p* < 0.01) and chl *a* content (*p* < 0.01) (Figure 6c). Moreover, ammonium concentration (*p* < 0.05), nitrate concentration (*p* < 0.05), silicate concentration (*p* < 0.05) and phosphate concentration (*p* < 0.05) played significant roles in shaping the *Nitrospira* communities (*p* < 0.05). For the *Nitrospira* communities, the key influential environmental factors in the ECS differed from those in the YS and the BS (Figure 6d). Specifically, in the BS and the YS, the *Nitrospira* communities were primarily influenced by ammonium concentration (*p* < 0.01) and chl *a* content (*p* < 0.01); however, salinity (*p* < 0.01) and temperature (*p* < 0.01) played more significant roles in the *Nitrospira* of the ECS.

### 3.4. Community Assembly of Nitrospira

**Figure 7:**
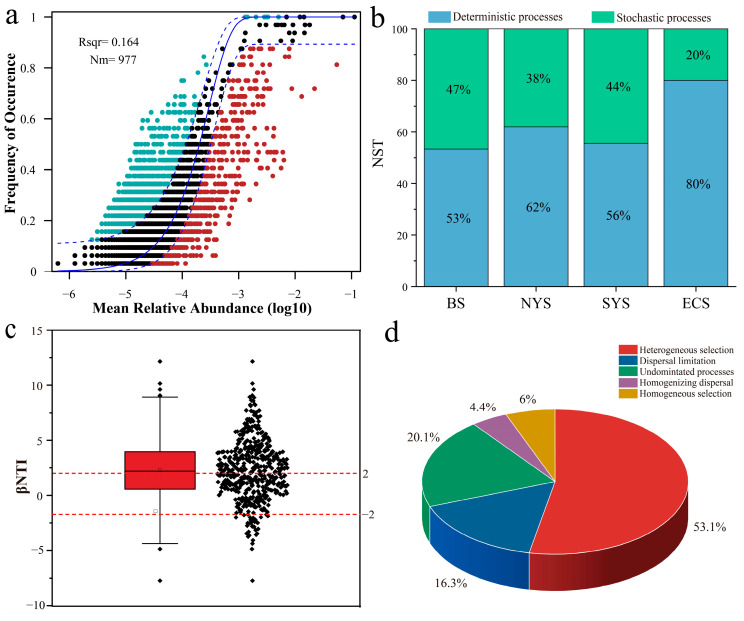
(**a**) Neutral community model of *Nitrospira* communities in sediments of the ECMSs. Blue and red dots indicate the OTUs that occur more and less frequently than predicted by the model. Dashed blue lines represent 95% confidence intervals around the model prediction. (**b**) Normalized stochasticity ratio of *Nitrospira* communities in sediments of the ECMSs. (**c**) βNTI analysis of *Nitrospira* communities in sediments of the ECMSs. The area between horizontal dashed lines (βNTI values of 2 and -2) indicates dominance of stochastic processes in community assembly, while values outside these thresholds represent deterministic process dominance. (**d**) Relative contributions of each processes on the assembly of *Nitrospira* communities in sediments of the ECMSs.

The NCM analysis reveals a weak correlation (R^2^ = 0.164) between the relative abundance and occurrence frequency of *Nitrospira* taxa, suggesting that stochastic processes might not be the main drivers of their community assembly (Figure 7a). Most NST values were below 50% across different seas, which indicates that deterministic processes dominantly influenced the community assembly of *Nitrospira*, with this influence being most pronounced in the ECS, followed by the NYS, the SYS and the BS (Figure 7b). The βNTI analysis shows that the majority of βNTI values exceeded 2, indicating the crucial role of deterministic processes in their community assembly in sediments of the ECMSs, which confirms the results of the NCM and NST analyses (Figure 7c). Deterministic processes accounted for 59.1% of the community assembly, with heterogeneous selection contributing 53.1% and homogeneous selection accounting for 6.0% (Figure 7d). However, stochastic processes had a lesser impact on the assembly of *Nitrospira* communities, with undominated processes and dispersal limitation accounting for 20.1% and 16.3%, respectively (Figure 7d). Thus, deterministic processes, especially heterogeneous selection processes, dominantly affected the community assembly of *Nitrospira* in sediments of the ECMSs.

### 3.5. Community Interaction and Stability of Nitrospira

**Figure 8:**
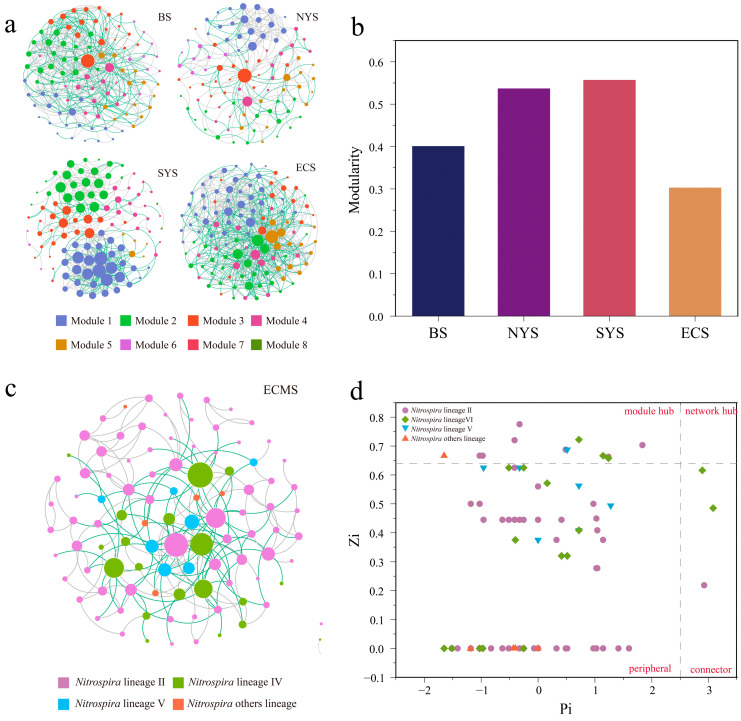
(**a**) Co-occurrence network analysis of the *Nitrospira* communities colored by modularity. (**b**) Network modularity index of the *Nitrospira* communities across the ECMSs. (**c**) Co-occurrence network analysis of the *Nitrospira* communities colored by lineage. (**d**) Zi-Pi plot of the *Nitrospira* communities in sediments of the ECMSs.

A co-occurrence network analysis was performed to explore the interactions within the *Nitrospira* communities in this study. The network of the ECS had the greatest number of nodes and edges, followed by the BS, the SYS and the NYS, indicating a more complex *Nitrospira* community in the ECS (Figure 8a). The network of the SYS was more modularized than other seas, suggesting a higher level of connectivity within the *Nitrospira* community in the SYS (Figure 8b). Lineage II played a dominant role in community interactions both with other *Nitrospira* taxa and among itself, which constituted over 65% of all interactions in this study (Figure 8c). Among the ninety-seven OTUs observed in this study, twelve OTUs (seven from lineage II, three from lineage IV, one from lineage V and one from other lineages) were identified as module hubs due to their high connectivity, making them key members within the *Nitrospira* communities (Figure 8d). In addition, three OTUs (one from lineage II and two from lineage IV) were classified as connectors to facilitate the interactions between different modules within the network. Overall, the results emphasize the essential role of lineage II in the interactions within the *Nitrospira* communities in sediments of the ECMSs.

**Figure 9:**
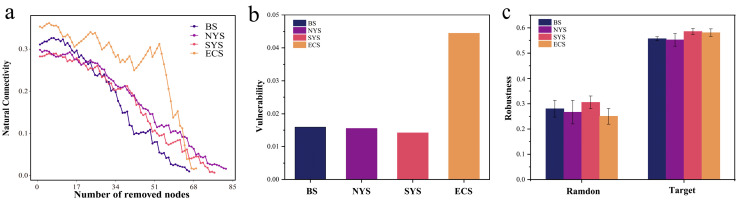
(**a**) The fragmentation of network with random removal of certain nodes. (**b**) Vulnerability of the *Nitrospira* communities. (**c**) Random (with the random removal of 50% of the nodes in the network) and target (with the removal of five key nodes in the network) robustness analysis of the *Nitrospira* communities.

Upon the random removal of certain nodes, the fragmentation of the *Nitrospira* community varied across seas. The community showed the highest degree of fragmentation in the SYS, followed by the NYS and the BS, while the *Nitrospira* community had the lowest degree of fragmentation in the ECS (Figure 9a). Furthermore, the *Nitrospira* community in the SYS showed lower vulnerability and higher robustness compared to other seas (Figure 9b,c). These results indicate that *Nitrospira* possessed the highest network stability in the SYS while presenting the lowest network stability under disturbances in the ECS.

## 4. Discussion

In this study, significant variations in the community composition and diversity of *Nitrospira* were observed across the ECMSs. The NMDS (Figure 3c) and phylogenetic analyses (Figure 5) reveal notable spatial heterogeneity among *Nitrospira* communities in different seas, with the BS and the NYS being the most similar, while the ECS exhibited the most distinct differences compared to other seas. Geographical distance was one of the primary drivers for microbial distribution patterns, as supported by our findings [44]. According to previous studies, most sediments in the BS, the NYS and the SYS (north of 33°N) primarily derive from the Yellow River, whereas most sediments in the SYS (south of 33°N) and the ECS mainly originate from the Yangtze River [45]. This difference in sediment sources likely contributes to the spatial heterogeneity of *Nitrospira* communities in the ECMSs, especially between the ECS and other seas.

In addition to geographical distances, environmental factors also influenced the distribution patterns of *Nitrospira* communities in this study. Similarly, the key influential environmental factors in the ECS differed from those in other seas (Figure 6d). Salinity and temperature were crucial in explaining the community variations across the studied seas (Figure 6c). Salinity, in particular, has been identified as one of the most important factors affecting *Nitrospira* communities, consistent with our results [6,46,47]. Previous research has shown that different *Nitrospira* taxa exhibit different tolerance to salinity. For example, *Nitrospira* lineage II is better adapted to regions with relatively low salinity, and lineage IV is more suitable for regions with relatively high salinity [47,48]. In our study, salinity in the ECS (34.68–33.37) was significantly higher than that in the YS (33.62–30.95) and the BS (30.63–29.26) (*p* < 0.05), which could explain why lineage II predominated in the BS and the YS, but lineage IV outcompeted lineage II in the ECS (Figure 4). The optimal temperature for *Nitrospira* growth in marine sediments ranges from 10 °C to 17 °C [49,50]. In line with these findings, the SYS (mean temperature: 11.9 °C) consistently showed the highest *Nitrospira* diversity across stations, whereas the NYS (8.9 °C) and BS (18.3 °C) exhibited relatively lower diversity. Notably, the ECS showed the highest temperature (20.7 °C), which displayed the lowest average diversity.

Most research on community assembly mechanisms has traditionally focused on whole microbiomes in diverse environments, while knowledge on the community assembly of specific functional microbial groups remains limited [51,52]. Our findings indicate that deterministic processes, rather than stochastic processes, shaped the community assembly of *Nitrospira* in these sediments. The coastlines of the ECMSs are crucial areas for economic development, where human activities, fisheries and tourism exert considerable pressure on the marine environment, leading to marine pollution and biodiversity decline [53,54]. Meanwhile, influenced by monsoons and ocean currents, the temperature and salinity of seawater exhibit significant seasonal fluctuations in these seas [55]. *Nitrospira* continuously adapts to its community structure in response to these complex and dynamic environmental changes. As a result, deterministic processes, particularly heterogeneous selection, made essential contributions to the community assembly of *Nitrospira* in this region. NST analysis reveals that the relative contribution of deterministic processes varied across the ECMSs, with a higher proportion in the ECS and the NYS (Figure 7b). Environmental factors, such as salinity, played important roles in governing the community assembly of *Nitrospira* in these sediments. High salinity often reduces nutrient utilization efficiency, making the community assembly of *Nitrospira* more susceptible to deterministic processes [56,57]. Our study shows that the highest relative contribution of deterministic processes was observed in the ECS, where the highest average salinity was observed. This finding aligns with observations in saline soils, where increased salinity corresponds with a greater relative contribution of deterministic processes in shaping *Nitrospira* communities [46]. Additionally, sediment input is frequently associated with microbial community migration. The BS is influenced by sediment discharge from the Haihe and the Yellow River basins, while the SYS receives sediment input from both the Yellow and Yangtze Rivers. Hence, compared to relatively uniform sediment sources of the NYS and the ECS, deterministic processes in the BS and the SYS may have a relatively lower contribution to community assembly.

Our research reveals complex interactions within *Nitrospira* in sediments of the ECMSs, with lineage II exhibiting notable dominance in the co-occurrence network. Lineage II accounted for over 65% of interactions, both across the whole region and within individual seas, and occupied a crucial central position within networks. Previous studies have shown that *Nitrospira* lineage II is able to thrive in a variety of complex and variable environments and can adapt to a wide range of salinity [46,47,58]. Therefore, it occupies an important ecological niche in marine sediments. The significant differences in network complexity among *Nitrospira* communities from different seas likely reflect their distinct environmental adaptability and resource utilization [59]. Robustness and vulnerability analyses of *Nitrospira* show that its stability differed across different seas. Temperature is a crucial factor influencing the stability of microbial interactions, as supported by our findings [60,61]. *Nitrospira* shows an optimal growth temperature ranging from 10 °C to 17 °C in marine environments [49,50]. The SYS, characterized by an average temperature within this optimal growth range, exhibited the highest level of community stability. In contrast, the ECS, where the average temperature exceeded the optimal growth temperature by the largest margin, displayed the lowest community stability. Additionally, sediment migration can impact microbial recovery capacity and enhance community stability through secondary colonization after disturbances [62]. Therefore, the differences in sediment sources between the ECS and the other two seas significantly contribute to variations in community stability. Furthermore, internal factors within the microbial community are also essential in explaining the differences in community stability. Some studies have suggested that high biodiversity and intense interactions may lead to instability within marine microbial communities [63,64]. In this study, interactions of *Nitrospira* in the ECS exhibited the highest complexity, which may be another important factor contributing to its lower community stability.

## 5. Conclusions

In this study, we conducted a comprehensive and systematic investigation into *Nitrospira* community characteristics in sediments of the ECMSs. The results reveal that community diversity, composition, complexity and stability differed across seas, implying a spatial distribution of *Nitrospira* communities in the ECMSs. Deterministic processes predominantly governed their community assembly. A great diversity of *Nitrospira* taxa was observed, with lineage II notably dominating their community in this study. Temperature, salinity, dissolved oxygen concentration, dissolved inorganic nutrient concentration and geographical distances significantly impacted the *Nitrospira* communities in sediments of the ECMSs. This study further enhances our understanding of the microbially driven nitrogen cycle in marine sediments and offers valuable insights into the adaptive strategies of marine microorganisms in response to environmental changes. Future research should focus on the impact of seasonal dynamics and sediment stratification on *Nitrospira* community characteristics, along with the assessment of their functional roles, to deepen our comprehension of the nitrogen cycle in marine sediments.

## Data Availability

The raw sequencing data generated in this study have been deposited in the NCBI Sequence Read Archive (SRA) database under accession numbers PRJNA1175424.
